# Mycotoxins – Determination of aflatoxins, ochratoxin A, free ochratoxin α, gliotoxin, citrinin, and dihydrocitrinone in urine by LC-MS/MS: Biomonitoring Method – Translation of the German version from 2025

**DOI:** 10.34865/bi116265e10_2or

**Published:** 2025-06-30

**Authors:** Marion Berger, Max Deharde, Judith Neuhoff, Bernhard Monien, Solveigh Siodlaczek, Thomas Göen, Andrea Hartwig, MAK Commission

**Affiliations:** 1 Federal Institute for Occupational Safety and Health (BAuA). Division 4 – Hazardous Substances and Biological Agents. Unit 4.2 – Health Surveillance. Biological Monitoring Nöldnerstraße 40/42 10317 Berlin Germany; 2 German Federal Institute for Risk Assessment. Unit 54 – Food Safety Department Max-Dohrn-Straße 8–10 10589 Berlin Germany; 3 Friedrich-Alexander-Universität Erlangen-Nürnberg. Institute and Outpatient Clinic of Occupational, Social, and Environmental Medicine Henkestraße 9–11 91054 Erlangen Germany; 4 Institute of Applied Biosciences. Department of Food Chemistry and Toxicology. Karlsruhe Institute of Technology (KIT) Adenauerring 20a, Building 50.41 76131 Karlsruhe Germany; 5 Permanent Senate Commission for the Investigation of Health Hazards of Chemical Compounds in the Work Area. Deutsche Forschungsgemeinschaft, Kennedyallee 40, 53175 Bonn, Germany. Further information: Permanent Senate Commission for the Investigation of Health Hazards of Chemical Compounds in the Work Area | DFG

**Keywords:** Mykotoxine, Aflatoxine, Ochratoxin A, Gliotoxin, Citrinin, Schimmelpilzgift, Biomonitoring, Urin, LC-MS/MS, mycotoxins, aflatoxins, ochratoxin A, gliotoxin, citrinin, mould toxin, biomonitoring, urine, LC-MS/MS

## Abstract

The working group “Analyses in Biological Materials” of the German Senate Commission for the Investigation of Health Hazards of Chemical Compounds in the Work Area (MAK Commission) developed and verified the presented biomonitoring method. The aim of this method is the selective and sensitive quantitation of aflatoxins (aflatoxins B1, B2, G1, G2, M1), ochratoxin A (OTA), free ochratoxin *α* (OT*α*), gliotoxin (GT), citrinin (CIT) and dihydrocitrinone (DH‑CIT) in urine. Sample preparation comprises enrichment and purification of the analytes by solid-phase extraction using OASIS HLB cartridges. Calibration is performed with comparative standards prepared in pooled urine and treated analogously to the samples to be analysed. The aflatoxins, OTA, CIT, and DH‑CIT are quantified using isotope-labelled internal standards (ISTDs), whereas OT*α* and GT are quantified without an ISTD. Determination is carried out by high-performance liquid chromatography-tandem mass spectrometry (LC‑MS/MS). The method provides reliable and accurate analytical results, as shown by the good precision data with standard deviations below 9% for the aflatoxins, OT*α*, GT and CIT, below 13% for OTA, and below 20% for DH‑CIT. Good accuracy data were obtained with mean relative recoveries in the range of 93–107% for the aflatoxins, OT*α*, GT and CIT, in the range of 83–103% for OTA, and in the range of 81–108% for DH‑CIT. The method is both selective and sensitive, and has quantitation limits in the range of 0.013–0.022 μg/l for the aflatoxins and OTA and a quantitation limit of 1.0 μg/l for OT*α*, 1.5 μg/l for GT, 0.0075 μg/l for CIT, and 0.01 μg/l for DH‑CIT.

## 1 Characteristics of the method

**Table:** 

**Matrix**	Urine		
**Analytical principle**	Liquid chromatography with tandem mass spectrometry (LC‑MS/MS)		
**Parameters and corresponding hazardous substances**			
**Hazardous substance**	**CAS No.**	**Parameter**	**CAS No.**
Aflatoxin B1 (AFB1)	1162-65-8	Aflatoxin B1 (AFB1)	1162-65-8
		Aflatoxin M1 (AFM1)	6795-23-9
Aflatoxin B2 (AFB2)	7220-81-7	Aflatoxin B2 (AFB2)	7220-81-7
Aflatoxin G1 (AFG1)	1165-39-5	Aflatoxin G1 (AFG1)	1165-39-5
Aflatoxin G2 (AFG2)	7241-98-7	Aflatoxin G2 (AFG2)	7241-98-7
Ochratoxin A (OTA)	303-47-9	Ochratoxin A (OTA)	303-47-9
		Ochratoxin *α* (OTα)	19165-63-0
Gliotoxin (GT)	67-99-2	Gliotoxin (GT)	67-99-2
Citrinin^a)^ (CIT)	518-75-2	Citrinin^a)^ (CIT)	518-75-2
		Dihydrocitrinone^a)^ (DH‑CIT)	65718-85-6

a) Information on the determination of citrinin and dihydrocitrinone can be found in the Appendix.

### Reliability criteria

#### Aflatoxin B1 (AFB1)

**Table:** 

Within-day precision:	Standard deviation (rel.)	*s_w_* = 2.9% or 1.8%
	Prognostic range	*u* = 7.4% or 4.6%
	at a spiked concentration of 0.0375 μg or 0.13 μg AFB1 per litre of urine and n = 6 determinations	
Day-to-day precision:	Standard deviation (rel.)	*s_w_* = 5.2% or 3.6%
	Prognostic range	*u* = 13.4% or 8.6%
	at a spiked concentration of 0.0375 μg or 0.13 μg AFB1 per litre of urine and n = 6 or 8 determinations	
Within-day accuracy:	Recovery (rel.)	*r* = 98.8% or 100%
	at a spiked concentration of 0.0375 μg or 0.13 μg AFB1 per litre of urine and n = 6 determinations	
Day-to-day accuracy:	Recovery (rel.)	*r* = 106% or 93.5%
	at a spiked concentration of 0.0375 μg or 0.13 μg AFB1 per litre of urine and n = 6 or 8 determinations	
Limit of detection:	0.004 μg AFB1 per litre of urine	
Limit of quantitation:	0.013 μg AFB1 per litre of urine	

#### Aflatoxin B2 (AFB2)

**Table:** 

Within-day precision:	Standard deviation (rel.)	*s_w_* = 2.4% or 2.2%
	Prognostic range	*u* = 6.2% or 5.6%
	at a spiked concentration of 0.0654 μg or 0.22 μg AFB2 per litre of urine and n = 6 determinations	
Day-to-day precision:	Standard deviation (rel.)	*s_w_* = 2.2% or 3.6%
	Prognostic range	*u* = 5.6% or 8.5%
	at a spiked concentration of 0.0654 μg or 0.22 μg AFB2 per litre of urine and n = 6 or 8 determinations	
Within-day accuracy:	Recovery (rel.)	*r* = 104% or 102%
	at a spiked concentration of 0.0654 μg or 0.22 μg AFB2 per litre of urine and n = 6 determinations	
Day-to-day accuracy:	Recovery (rel.)	*r* = 103% or 95.7%
	at a spiked concentration of 0.0654 μg or 0.22 μg AFB2 per litre of urine and n = 6 or 8 determinations	
Limit of detection:	0.007 μg AFB2 per litre of urine	
Limit of quantitation:	0.022 μg AFB2 per litre of urine	

#### Aflatoxin G1 (AFG1)

**Table:** 

Within-day precision:	Standard deviation (rel.)	*s_w_* = 3.5% or 2.6%
	Prognostic range	*u* = 9.1% or 6.8%
	at a spiked concentration of 0.0375 μg or 0.13 μg AFG1 per litre of urine and n = 6 determinations	
Day-to-day precision:	Standard deviation (rel.)	*s_w_* = 4.9% or 3.5%
	Prognostic range	*u* = 12.5% or 8.3%
	at a spiked concentration of 0.0375 μg or 0.13 μg AFG1 per litre of urine and n = 6 or 8 determinations	
Within-day accuracy:	Recovery (rel.)	*r* = 99.4% or 100%
	at a spiked concentration of 0.0375 μg or 0.13 μg AFG1 per litre of urine and n = 6 determinations	
Day-to-day accuracy:	Recovery (rel.)	*r* = 107% or 94.6%
	at a spiked concentration of 0.0375 μg or 0.13 μg AFG1 per litre of urine and n = 6 or 8 determinations	
Limit of detection:	0.004 μg AFG1 per litre of urine	
Limit of quantitation:	0.013 μg AFG1 per litre of urine	

#### Aflatoxin G2 (AFG2)

**Table:** 

Within-day precision:	Standard deviation (rel.)	*s_w_* = 2.0% or 3.2%
	Prognostic range	*u* = 5.0% or 8.3%
	at a spiked concentration of 0.163 μg or 0.54 μg AFG2 per litre of urine and n = 6 determinations	
Day-to-day precision:	Standard deviation (rel.)	*s_w_* = 2.7% or 3.3%
	Prognostic range	*u* = 7.0% or 7.8%
	at a spiked concentration of 0.163 μg or 0.54 μg AFG2 per litre of urine and n = 6 or 8 determinations	
Within-day accuracy:	Recovery (rel.)	*r* = 100% or 103%
	at a spiked concentration of 0.163 μg or 0.54 μg AFG2 per litre of urine and n = 6 determinations	
Day-to-day accuracy:	Recovery (rel.)	*r* = 95.8% or 96.3%
	at a spiked concentration of 0.163 μg or 0.54 μg AFG2 per litre of urine and n = 6 or 8 determinations	
Limit of detection:	0.02 μg AFG2 per litre of urine	
Limit of quantitation:	0.054 μg AFG2 per litre of urine	

#### Aflatoxin M1 (AFM1)

**Table:** 

Within-day precision:	Standard deviation (rel.)	*s_w_* = 3.1% or 3.6%
	Prognostic range	*u* = 7.9% or 9.4%
	at a spiked concentration of 0.066 μg or 0.22 μg AFM1 per litre of urine and n = 6 determinations	
Day-to-day precision:	Standard deviation (rel.)	*s_w_* = 3.6% or 5.9%
	Prognostic range	*u* = 9.3% or 13.9%
	at a spiked concentration of 0.066 μg or 0.22 μg AFM1 per litre of urine and n = 6 or 8 determinations	
Within-day accuracy:	Recovery (rel.)	*r* = 108% or 101%
	at a spiked concentration of 0.066 μg or 0.22 μg AFM1 per litre of urine and n = 6 determinations	
Day-to-day accuracy:	Recovery (rel.)	*r* = 103% or 96.4%
	at a spiked concentration of 0.066 μg or 0.22 μg AFM1 per litre of urine and n = 6 or 8 determinations	
Limit of detection:	0.007 μg AFM1 per litre of urine	
Limit of quantitation:	0.022 μg AFM1 per litre of urine	

#### Ochratoxin A (OTA)

**Table:** 

Within-day precision:	Standard deviation (rel.)	*s_w_* = 1.5% or 3.6%
	Prognostic range	*u* = 3.9% or 9.4%
	at a spiked concentration of 0.0377 μg or 0.13 μg OTA per litre of urine and n = 6 determinations	
Day-to-day precision:	Standard deviation (rel.)	*s_w_* = 8.8% or 12.5%
	Prognostic range	*u* = 22.7% or 30.6%
	at a spiked concentration of 0.0377 μg or 0.13 μg OTA per litre of urine and n = 6 or 7 determinations	
Within-day accuracy:	Recovery (rel.)	*r* = 103% or 93.5%
	at a spiked concentration of 0.0377 μg or 0.13 μg OTA per litre of urine and n = 6 determinations	
Day-to-day accuracy:	Recovery (rel.)	*r* = 92.5% or 82.9%
	at a spiked concentration of 0.0377 μg or 0.13 μg OTA per litre of urine and n = 6 or 7 determinations	
Limit of detection:	0.004 μg OTA per litre of urine	
Limit of quantitation:	0.013 μg OTA per litre of urine	

#### Ochratoxin α (OTα)

**Table:** 

Within-day precision:	Standard deviation (rel.)	*s_w_* = 1.3% or 4.0%
	Prognostic range	*u* = 3.3% or 10.4%
	at a spiked concentration of 2.5 μg or 6.25 μg OTα per litre of urine and n = 6 determinations	
Day-to-day precision:	Standard deviation (rel.)	*s_w_* = 5.2% or 6.5%
	Prognostic range	*u* = 13.2% or 15.3%
	at a spiked concentration of 2.5 μg or 6.25 μg OTα per litre of urine and n = 6 or 8 determinations	
Within-day accuracy:	Recovery (rel.)	*r* = 95.4% or 93.4%
	at a spiked concentration of 2.5 μg or 6.25 μg OTα per litre of urine and n = 6 determinations	
Day-to-day accuracy:	Recovery (rel.)	*r* = 99.9% or 99.5%
	at a spiked concentration of 2.5 μg or 6.25 μg OTα per litre of urine and n = 6 or 8 determinations	
Limit of detection:	0.4 μg OTα per litre of urine	
Limit of quantitation:	1.0 μg OTα per litre of urine	

#### Gliotoxin (GT)

**Table:** 

Within-day precision:	Standard deviation (rel.)	*s_w_* = 1.7% or 4.9%
	Prognostic range	*u* = 4.4% or 12.7%
	at a spiked concentration of 3.75 μg or 8.75 μg GT per litre of urine and n = 6 determinations	
Day-to-day precision:	Standard deviation (rel.)	*s_w_* = 6.6% or 8.6%
	Prognostic range	*u* = 16.9% or 20.4%
	at a spiked concentration of 3.75 μg or 8.75 μg GT per litre of urine and n = 6 or 8 determinations	
Within-day accuracy:	Recovery (rel.)	*r* = 95.4% or 92.9%
	at a spiked concentration of 3.75 μg or 8.75 μg GT per litre of urine and n = 6 determinations	
Day-to-day accuracy:	Recovery (rel.)	*r* = 97.1% or 104%
	at a spiked concentration of 3.75 μg or 8.75 μg GT per litre of urine and n = 6 or 8 determinations	
Limit of detection:	0.5 μg GT per litre of urine	
Limit of quantitation:	1.5 μg GT per litre of urine	

#### Citrinin (CIT)^a)^

**Table:** 

Within-day precision:	Standard deviation (rel.)	*s_w_* = 3.1%, 6.0% or 6.5%
	Prognostic range	*u* = 9.4%, 16.5% or 17.2%
	at a spiked concentration of 0.01 μg, 0.1 μg or 1.0 μg CIT per litre of urine and n = 6 determinations	
Day-to-day precision:	Standard deviation (rel.)	*s_w_* = 6.4%, 5.9% or 2.1%
	Prognostic range	*u* = 17.5%, 15.2% or 6.2%
	at a spiked concentration of 0.01 μg, 0.1 μg or 1.0 μg CIT per litre of urine and n = 6 determinations	
Within-day accuracy:	Recovery (rel.)	*r* = 94.1%, 88.6% or 93.3%
	at a spiked concentration of 0.01 μg, 0.1 μg or 1.0 μg CIT per litre of urine and n = 6 determinations	
Day-to-day accuracy:	Recovery (rel.)	*r* = 99.8%, 99.0% or 98.5%
	at a spiked concentration of 0.01 μg, 0.1 μg or 1.0 μg CIT per litre of urine and n = 6 determinations	
Limit of detection:	0.0003 μg CIT per litre of urine	
Limit of quantitation:	0.001 μg CIT per litre of urine	

a) These data were collected by the verifiers of the method. Information on the determination of citrinin and dihydrocitrinone can be found in the Appendix.

#### Dihydrocitrinone (DH‑CIT)^a)^

**Table:** 

Within-day precision:	Standard deviation (rel.)	*s_w_* = 14.0%, 3.3% or 8.9%
	Prognostic range	*u* = 37.5%, 10.1% or 23.9%
	at a spiked concentration of 0.01 μg, 0.1 μg or 1.0 μg DH-CIT per litre of urine and n = 6 determinations	
Day-to-day precision:	Standard deviation (rel.)	*s_w_* = 19.3%, 5.4% or 3.4%
	Prognostic range	*u* = 54.7%, 12.1% or 9.9%
	at a spiked concentration of 0.01 μg, 0.1 μg or 1.0 μg DH-CIT per litre of urine and n = 6 determinations	
Within-day accuracy:	Recovery (rel.)	*r* = 108%, 80.9% or 88.5%
	at a spiked concentration of 0.01 μg, 0.1 μg or 1.0 μg DH-CIT per litre of urine and n = 6 determinations	
Day-to-day accuracy:	Recovery (rel.)	= 108%, 88.4% or 96.4%
	at a spiked concentration of 0.01 μg, 0.1 μg or 1.0 μg DH-CIT per litre of urine and n = 6 determinations	
Limit of detection:	0.0075 μg DH-CIT per litre of urine	
Limit of quantitation:	0.01 μg DH-CIT per litre of urine	

a) These data were collected by the verifiers of the method. Information on the determination of citrinin and dihydrocitrinone can be found in the Appendix.

## 2 General information on the mycotoxins

**Fig.1:**
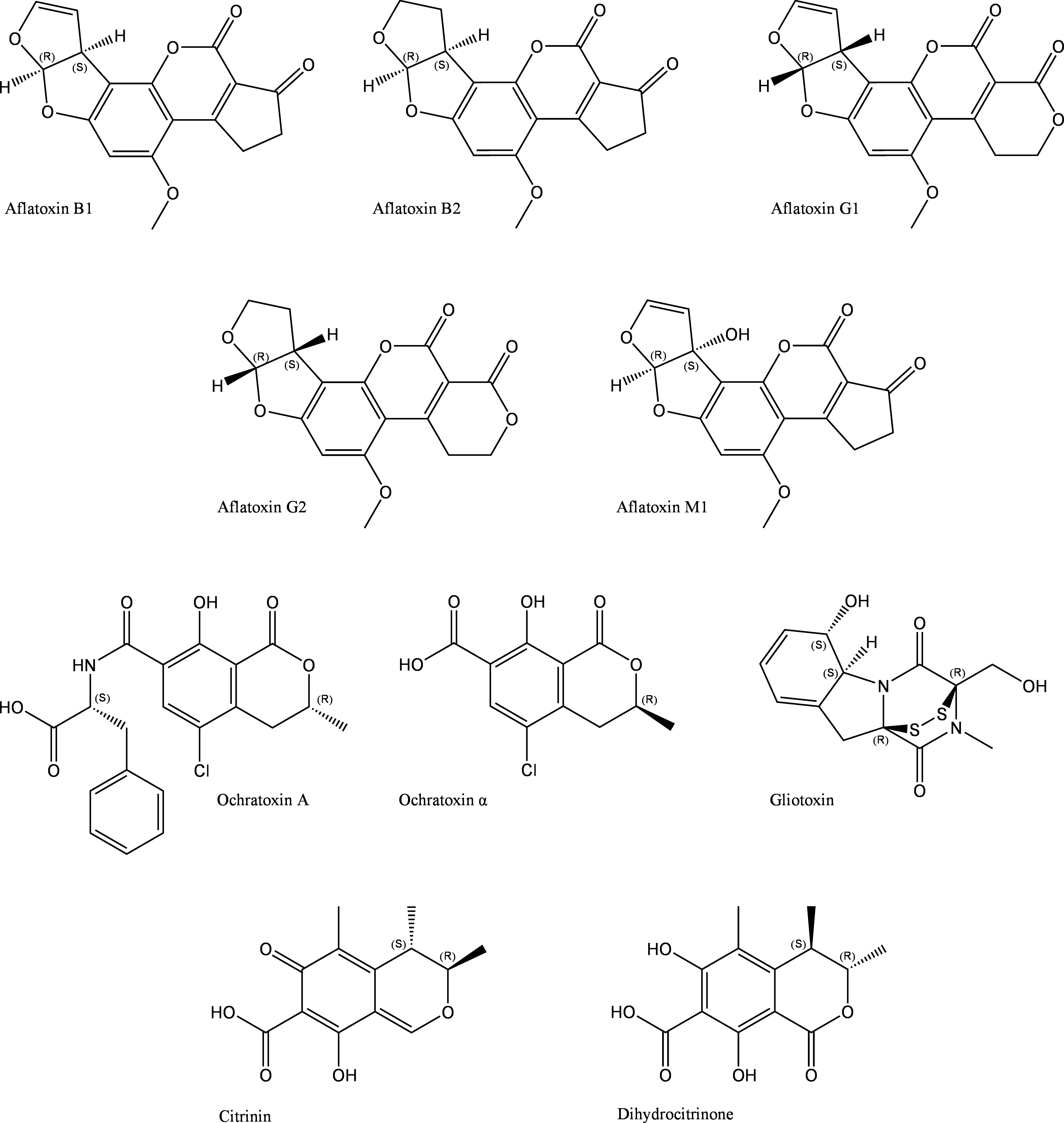
Structural formulas of aflatoxin B1, aflatoxin B2, aflatoxin G1, aflatoxin G2, aflatoxin M1, ochratoxin A, ochratoxin α, gliotoxin, citrinin, and dihydrocitrinone

The structural formulas of the mycotoxins and mycotoxin metabolites that can be quantified using this method are shown in Figure 1. A method for the determination of further mycotoxins in urine (deoxynivalenol and deepoxydeoxy­nivalenol) was published by the Commission (Berger et al. 2025).

### Aflatoxins

The naturally occurring aflatoxins AFB1, AFB2, AFG1, and AFG2 are bisfuranocoumarin compounds which are produced by various fungi of the *Aspergillus* genus, particularly *A. flavus* and *A. parasiticus* (CONTAM et al. 2020).

In humans, aflatoxins are genotoxic and carcinogenic (CONTAM et al. 2020). The Commission has classified aflatoxins in Carcinogen Category 1 and in Germ Cell Mutagen Category 3 A (Greim 2008).

Aflatoxins can be ingested orally or inhaled (Greim 2008). Moreover, *in vitro* experiments show a slight ability for AFB1 to penetrate the skin (Boonen et al. 2012). Toxicokinetic data for humans are only available for AFB1. After oral uptake, AFB1 is rapidly absorbed via the gastrointestinal tract, whereby maximum concentrations in the blood plasma are reached within one hour (Jubert et al. 2009). Two-phase kinetics are observed for AFB1: a rapid distribution and excretion phase with a plasma half-life of around 2.9 h is followed by a slow excretion phase with a plasma half-life of approx. 64 h. 95% of AFB1 excreted via the urine is excreted within one day (Jubert et al. 2009). Aflatoxins are primarily metabolised in the liver (Al-Jaal et al. 2019). Four main metabolic pathways are known for AFB1: demethylation to aflatoxin P1, ketoreduction to aflatoxicol, hydroxylation to AFM1, and epoxidation to the AFB1-8,9‑epoxide (Dohnal et al. 2014). The 8,9‑epoxides of AFB1 and AFG1, which are formed by oxidation of the furan double bond, can react with DNA and other nucleophiles (CONTAM et al. 2020). AFB1 thereby forms covalent DNA adducts with N7‑guanine and causes DNA lesions (CONTAM et al. 2020).

The general population is exposed to aflatoxins via the consumption of contaminated food products (e.g. of peanuts and spices) (CONTAM et al. 2020). Occupational exposure has been described in agriculture and food production as well as in waste disposal (Brera et al. 2002; Ferri et al. 2017; Fromme et al. 2016; Viegas et al. 2013, 2015, 2018 a).

In addition to the aflatoxins (AFB1, AFB2, AFG1, and AFG2), the metabolite AFM1 and the guanine adduct 8,9‑di­hydro-8‑(N7‑guanyl)-9‑hydroxy-AFB1 (AFB1-N7‑Gua) are used as urinary biomarkers for aflatoxin exposure (Al-Jaal et al. 2019). AFM1 and AFB1-N7‑Gua are recognised as valid biomarkers of recent aflatoxin exposure (CONTAM et al. 2020). Due to the availability of analytical standards, AFM1 is more frequently used as biomarker (Martins et al. 2021).

### Ochratoxin A (OTA)

OTA is formed by fungi of the *Aspergillus* and *Penicillium* genera and possesses carcinogenic, nephrotoxic, reprotoxic, and immunotoxic properties (Tao et al. 2018). The Commission has classified OTA in Carcinogen Category 2 and in Germ Cell Mutagen Category 3 B (Greim 2006).

Orally ingested OTA is well absorbed via the gastrointestinal tract and subsequently almost completely bound to serum proteins, such as albumin. At 0.02%, the proportion of unbound OTA in the blood is rather small (Hagelberg et al. 1989).

In humans, OTA is primarily excreted via the kidneys. Due to the strong protein binding, glomerular filtration only takes place to a limited extent. OTA instead reaches the urine via tubular secretion. OTA is reabsorbed in all segments of the nephron, whereby accumulation can occur in the kidneys (HBM-Kommission 2014).

Biphasic toxicokinetics are observed in both animal and human studies (data from one individual), whereby a rapid distribution and excretion phase (plasma half-life of about 20 h) is followed by a slow excretion phase with a plasma half-life of about 35 days (Studer-Rohr et al. 2000). About 50% of absorbed OTA is excreted unmetabolised. OTA is metabolised in the kidneys, liver, and intestines. The main metabolite is OTα, which is partly present as glucuronide and/or sulfate ester (Muñoz et al. 2010).

The general population is exposed to OTA via the consumption of contaminated food products (e.g. of coffee and grain products). Occupational exposure has been described in agriculture and food production (Brera et al. 2002; Fromme et al. 2016; Viegas et al. 2019) as well as in waste disposal (Degen et al. 2003; Viegas et al. 2018 b).

Due to its long plasma half-life, chronic exposures (over several weeks) are determined via OTA concentrations in serum/plasma. Urinary OTA concentrations, on the other hand, better reflect acute exposures (Duarte et al. 2011; EFSA 2006).

### Gliotoxin (GT)

The epipolythiodioxopiperazine GT is a sulfur-containing mycotoxin which is formed by various mould fungi, including *Aspergillus fumigatus*, *Eurotium chevalieri*, *Trichoderma virens*, and *Neosartorya pseudofischeri* (Scharf et al. 2016). GT is immunosuppressive, genotoxic, and cytotoxic (Nieminen et al. 2002 a, b). Furthermore, it is suspected of having an influence on the virulence of *A. fumigatus* and may, in turn, promote the formation of an invasive aspergillosis (Hof and Kupfahl 2009). Gliotoxin has not yet been evaluated by the Commission.

There are almost no data on the toxicokinetics and metabolism of the substance in the human body. De Santis et al. (2017) were able to determine GT in 71.6% of children’s urine samples with a maximum concentration of 114.7 μg/l. Moreover, the use of GT as a biomarker in serum and urine samples for the early detection of invasive aspergillosis has been discussed (Cerqueira et al. 2014; Gao et al. 2019).

High concentrations of the ubiquitously occurring *A. fumigatus* are observed, for example, in bioaerosol samples from biowaste treatment (Fischer et al. 2000). Only few studies have investigated GT concentrations in air and dust samples of the agricultural sector. In dust samples from granaries, GT concentrations of up to 319.6 μg/g were determined (Tangni and Pussemier 2007). Lanier et al. (2010) report on stable-air concentrations of up to 3.7 μg/m^3^ when feeding corn silage, hay, and oil seeds.

Systematic investigations on GT contamination in food products or on dietary background exposure are not available (Scharf et al. 2016).

### Citrinin (CIT)

CIT is a polyketide mycotoxin that is a secondary metabolite produced by fungi and is named after *Penicillium citrinum*, from which it was first isolated. CIT is produced by several species of the genera *Penicillium*, *Aspergillus* and *Monascus* and can be detected in different climate zones (CONTAM 2012).

CIT is often found along with the structurally and toxicologically similar OTA. The kidneys are the primary target organ of both mycotoxins in several mammalian species (CONTAM 2012; EFSA 2006). The International Agency for Research on Cancer (IARC) classified CIT as a group 3 compound (not classifiable as to its carcinogenicity to humans) due to suspected carcinogenicity in rats and insufficient evidence in humans (IARC 1986). The European Food Safety Authority (EFSA) could not draw any conclusions regarding the potential carcinogenicity of CIT (CONTAM 2012). CIT has not yet been evaluated by the Commission.

CIT is mainly found in stored grain and cereal-based products as well as in other mould-contaminated plant products such as fruits, herbs and spices. CIT is mainly formed during storage, whereby concentrations of up to 1500 μg/kg were detected (CONTAM 2012). Very high levels of CIT (> 2000 μg/kg) were found in red mould rice, which is used as a preservative and colouring agent in Asian foods and is marketed as a food supplement (Degen et al. 2022).

The main CIT exposure is via the diet, but also dermal (Boonen et al. 2012) and inhalative (Föllmann et al. 2016) uptake seems to be possible. The investigation of CIT kinetics in humans shows rapid absorption after oral uptake and a mean half-life for CIT of 9.4 h in plasma (Degen et al. 2018). CIT is largely metabolised to DH-CIT, which is excreted in the urine together with the unmetabolised compound. The average excretion half-life of CIT and DH-CIT in urine is 6.7 h and 8.9 h, respectively. Thereby, about 40% (sum of CIT and DH-CIT) of the ingested mycotoxin dose is excreted within 24 h (Degen et al. 2018).

## 3 General principles

The method described herein serves for the measurement of aflatoxins (AFB1, AFB2, AFG1, AFG2, AFM1), OTA, free OTα and GT in human urine by LC‑MS/MS. Sample preparation comprises purification of the samples by solid-phase extraction using OASIS HLB cartridges, followed by concentration of the eluates under a stream of nitrogen. Cali­bration is performed with comparative standards prepared in urine and treated analogously to the samples to be analysed. The aflatoxins are quantified using ^13^C_17_‑AFB1, OTA is quantified using ^13^C_20_‑OTA, whereas OTα and GT are quantified without using an ISTD.

During the external verification, citrinin and its metabolite DH-CIT were also integrated into the method. Information on the determination of these parameters and the corresponding validation data can be found in the Appendix.

## 4 Equipment, chemicals, and solutions

### 4.1 Equipment

- HPLC system with a binary pump, autosampler, column oven, and degasser (e.g. Nexera XR, Shimadzu Deutschland GmbH, Duisburg, Germany)
- Triple-quadrupole mass spectrometer (e.g. AB SCIEX QTRAP 5500, AB SCIEX Germany GmbH, Darmstadt, Germany)
- Analytical HPLC separation column (e.g. Kinetex^®^ Core-Shell technology; Kinetex^®^ 2.6 μm biphenyl 100 Å, 100 × 2.1 mm, Phenomenex Ltd. Deutschland, Aschaffenburg, Germany)
- UHPLC precolumn (e.g. No. AJO-9209, SecurityGuard ULTRA Cartridges, biphenyl 2.1 mm ID, including column holder, Phenomenex Ltd. Deutschland, Aschaffenburg, Germany)
- Nitrogen generator (e.g. cmc Instruments GmbH, Eschborn, Germany)
- Water-purification system (e.g. Veolia Water Solutions & Technologies, Saint-Maurice, France)
- Laboratory centrifuge (e.g. Fisher Scientific GmbH, Schwerte, Germany)
- Analytical balance (e.g. Sartorius AG, Göttingen, Germany)
- pH meter (e.g. Mettler-Toledo GmbH, Gießen, Germany)
- Evaporator (e.g. Biotage Sweden AB, Uppsala, Sweden)
- Ultrasonic bath for degassing the eluents (e.g. SONOREX SUPER RK 510 H, BANDELIN electronic GmbH & Co. KG, Berlin, Germany)
- Tube rotator (e.g. Cole-Parmer^TM^ Stuart^TM^, Fisher Scientific GmbH, Schwerte, Germany)
- Vortex shaker (e.g. IKA‑Werke GmbH & Co. KG, Staufen, Germany)
- SPE vacuum manifold (e.g. VisiPrep^TM^ SPE Vacuum Manifold, Supelco^®^, Merck KGaA, Darmstadt, Germany)
- Variably adjustable pipettes with matching pipette tips (e.g. Eppendorf AG, Hamburg, Germany)
- 2500‑ml glass bottles with screw cap (e.g. DURAN^®^, Schott AG, Mainz, Germany)
- Various volumetric flasks and glass beakers (e.g. DURAN^®^, Schott AG, Mainz, Germany)
- 10‑ml amber glass bottles (e.g. DURAN^®^, Schott AG, Mainz, Germany)
- 0.2‑μm syringe filter (13 mm, regenerated cellulose) (e.g. CS – Chromatographie Service GmbH, Langerwehe, Germany)
- SPE cartridges, OASIS^®^ HLB, 150 mg/6 ml (e.g. Waters GmbH, Eschborn, Germany)
- 15‑ml polypropylene conical centrifuge tubes (e.g. COTECH Vertriebs GmbH, Berlin, Germany)
- 13‑ml polypropylene round-bottom centrifuge tubes (e.g. COTECH Vertriebs GmbH, Berlin, Germany)
- 5‑ml disposable Luer-Lock syringes with disposable injection cannulae (e.g. Omnifix^®^ Luer Solo, B. Braun SE, Melsungen, Germany)
- 1.5‑ml polypropylene threaded vials with screw caps (e.g. MACHEREY-NAGEL GmbH & Co. KG, Düren, Germany)
- Urine cups made of polypropylene (e.g. Sarstedt AG & Co. KG, Nümbrecht, Germany)

### 4.2 Chemicals

Unless otherwise specified, all chemicals must be a minimum of *pro analysi* grade.

- Acetic acid, LiChropur^®^, 100% (e.g. No. 533001, Supelco^®^, Merck KGaA, Darmstadt, Germany)
- Acetonitrile, LC‑MS (e.g. No. 15037, Burdick & Jackson^TM^, Honeywell International Inc., Morristown, USA)
- Ammonium acetate (e.g. No. 15681570, Honeywell Fluka^TM^, Fisher Scientific GmbH, Schwerte, Germany)
- Hydrochloric acid, ROTIPURAN^®^, 37% (e.g. No. 4325.1, Carl Roth GmbH + Co. KG, Karlsruhe, Germany)
- Isopropanol, LiChrosolv^®^, for piston backwash (e.g. No. 102781, Supelco^®^, Merck KGaA, Darmstadt, Germany)
- Methanol, LiChrosolv^®^, ≥ 99.97% (e.g. No. 106035, Supelco^®^, Merck KGaA, Darmstadt, Germany)
- Ultra-pure water (e.g. Veolia Water Solutions & Technologies, Saint-Maurice, France)
- Native urine from volunteers with mycotoxin levels as low as possible

### 4.3 Reference standards and ISTDs

- Aflatoxin B1, 2 μg/ml in acetonitrile (e.g. Romer Labs Division Holding GmbH, Getzersdorf, Austria)
- ^13^C_17_‑Aflatoxin B1, 0.501 μg/ml in acetonitrile (e.g. No. DRE‑A10047150AL‑0.5, LGC Standards GmbH, Wesel, Germany)
- Aflatoxin B2 (e.g. No. A9887, Sigma-Aldrich^®^, Merck KGaA, Darmstadt, Germany)
- Aflatoxin G1, 2 μg/ml in acetonitrile (e.g. Romer Labs Division Holding GmbH, Getzersdorf, Austria)
- Aflatoxin G2, 0.51 μg/ml in acetonitrile (e.g. Romer Labs Division Holding GmbH, Getzersdorf, Austria)
- Aflatoxin M1, 0.506 μg/ml in acetonitrile (e.g. Romer Labs Division Holding GmbH, Getzersdorf, Austria)
- Ochratoxin A, 10.05 μg/ml in acetonitrile (e.g. Romer Labs Division Holding GmbH, Getzersdorf, Austria)
- ^13^C_20_‑Ochratoxin A, 10.10 μg/ml in acetonitrile (e.g. Romer Labs Division Holding GmbH, Getzersdorf, Austria)
- Gliotoxin (e.g. No. G9893, Sigma-Aldrich^®^, Merck KGaA, Darmstadt, Germany)
- Ochratoxin α, 10.2 μg/ml in acetonitrile (e.g. Romer Labs Division Holding GmbH, Getzersdorf, Austria)

### 4.4 Solutions

- Eluent A
  77.08 mg of ammonium acetate are weighed into a 1000‑ml volumetric flask and dissolved in a little ultra-pure water. Subsequently, 1 ml of acetic acid is pipetted into the flask, which is then made up to the mark with ultra-­pure water.
- Eluent B
  77.08 mg of ammonium acetate are weighed into a 1000‑ml volumetric flask and dissolved in a little methanol. Subsequently, 1 ml of acetic acid is pipetted into the flask, which is then made up to the mark with methanol.
- Gradient solution (Eluent A ∶ Eluent B; 98 ∶ 2 (v : v))
  2 ml of Eluent B are pipetted into a 100‑ml volumetric flask, which is then made up to the mark with Eluent A.
- Diluted hydrochloric acid (55 mM)
  Approx. 550 ml of ultra-pure water are placed in a 1000 ml volumetric flask and 4.56 ml of 37% hydrochloric acid are added. Subsequently, the volumetric flask is made up to the mark with ultra-pure water.
- Methanol (2% in ultra-pure water)
  2 ml of methanol are placed in a 100‑ml volumetric flask, which is then made up to the mark with ultra-pure water.

The solutions are stored at room temperature.

### 4.5 Internal standard (ISTD)

- ISTD spiking solution (30.12 μg ^13^C_17_‑AFB1/l and 30.3 μg ^13^C_20_‑OTA/l)
  60 μl of the ^13^C_17_‑AFB1 standard and 3 µl of the ^13^C_20_‑OTA standard are mixed with 937 μl of acetonitrile in a 1.5‑ml polypropylene vial.

The ISTD spiking solution is stored at −20 °C and must be freshly prepared every week.

### 4.6 Calibration standards

- AFB2 working solution I (100 mg/l)
  5 mg of AFB2 are weighed into a 50‑ml volumetric flask and 5 ml of methanol are added. The volumetric flask is then filled up to the mark with acetonitrile and AFB2 is dissolved by shaking.

The working solution I is portioned into 10‑ml amber glass bottles with plastic screw caps and stored at −20 °C.

- AFB2 working solution II (1 mg/l)
  10 μl of the AFB2 working solution I are added to 990 μl of acetonitrile and the solution is mixed.

The AFB2 working solution II is freshly prepared for each calibration as well as for the preparation of the quality-control samples.

- OTA working solution (2.01 mg/l)
  40 μl of the OTA stock solution (10.05 mg/l acetonitrile) are added to 160 μl of acetonitrile and the solution is thoroughly mixed.

The OTA working solution is freshly prepared for each calibration as well as for the preparation of the quality-control samples.

- GT working solution I (500 mg/l)
  5 mg of GT are weighed into a 10‑ml volumetric flask and dissolved in a little acetonitrile. Subsequently, the volumetric flask is made up to the mark with acetonitrile.

The GT working solution I is portioned into 1.5‑ml polypropylene vials and stored at −20 °C.

- GT working solution II (100 mg/l)
  200 μl of the GT working solution I are added to 800 μl of acetonitrile and the solution is thoroughly mixed in a 1.5‑ml polypropylene vial.

The GT working solution II is freshly prepared for each calibration as well as for the preparation of the quality-control samples.

- Spiking solution 1 (S 1)
  The spiking solution 1 is prepared in a 1.5‑ml polypropylene vial according to the pipetting scheme given in Table 1.

**Tab.1:** Pipetting scheme for the preparation of spiking solution 1

Analyte	Solution	Volume [μl]	Acetonitrile [μl]	Analyte concentration [μg/l]
AFB1	AFB1 stock solution	10	695	20
AFB2	AFB2 working solution II	35		35
AFG1	AFG1 stock solution	10		20
AFG2	AFG2 stock solution	170		86.7
AFM1	AFM1 stock solution	70		35.4
OTA	OTA working solution	10		20.1

- Spiking solution 2 (S 2; 500 μg OTα/l)
  50 μl of the OTα stock solution are mixed with 950 μl of methanol in a 1.5‑ml polypropylene vial.
- Spiking solution 3 (S 3; 1000 μg GT/l)
  10 μl of the GT working solution II are mixed with 990 μl of acetonitrile in a 1.5‑ml polypropylene vial.

The spiking solutions 1 to 3 are stored at −20 °C and must be freshly prepared every week.

The calibration standards are prepared in urine that is as uncontaminated as possible. To prepare the calibration standards, the spiking solutions 1 to 3 are pipetted into 4 ml of urine according to the pipetting scheme given in Table 2. The concentrations of the analytes in the respective calibration standards are listed in Table 3. The calibration solutions are prepared in the same way as the samples to be measured as described in Section 5, but without the addition of ISTD.

**Tab.2:** Pipetting scheme for the preparation of the calibration standards

Solution	S 1 [μl]	S 2 [μl]	S 3 [μl]	ISTD spiking solution [μl]
DB^a)^	0	0	0	0
B^b)^	0	0	0	20
1	2.5	8	6	20
2	5	16	12	20
3	10	24	18	20
4	15	32	24	20
5	20	40	30	20
6	30	60	40	20

a) double-blank

b) blank

**Tab.3:** Concentration of the analytes and the ISTDs in the calibration standards

Solution	Concentration [μg/l]									
	^13^C_17_-AFB1	^13^C_20_-OTA	AFB1	AFB2	AFG1	AFG2	AFM1	OTA	OTα	GT
DB^a)^	0	0	0	0	0	0	0	0	0	0
B^b)^	0.15	0.15	0	0	0	0	0	0	0	0
1	0.15	0.15	0.013	0.022	0.013	0.054	0.022	0.013	1	1.5
2	0.15	0.15	0.025	0.044	0.025	0.108	0.044	0.025	2	3
3	0.15	0.15	0.05	0.088	0.05	0.217	0.088	0.05	3	4.5
4	0.15	0.15	0.075	0.131	0.075	0.325	0.133	0.075	4	6
5	0.15	0.15	0.1	0.175	0.1	0.434	0.177	0.101	5	7.5
6	0.15	0.15	0.15	0.262	0.15	0.65	0.265	0.151	7.5	10

a) double-blank

b) blank

### 4.7 Control-standard solution

The measurement of a control-standard solution is used to test the equilibrated measurement system (testing for pressure, peak intensities, and retention times). The control standard is analysed at the beginning and at the end of a run.

20 μl of each of the spiking solutions 1 to 3 and 20 μl of the ISTD spiking solution are mixed with 920 μl of the gradient solution in a 1.5‑ml polypropylene vial to prepare the control-standard solution. The solution is stored at −20 °C and freshly prepared every week. Table 4 shows the concentrations of the analytes and the ISTDs in the control-standard solution.

**Tab.4:** Concentrations of analytes and ISTDs in the control-standard solution

Analyte/ISTD	Concentration [μg/l]
AFB1	0.4
AFB2	0.7
AFG1	0.4
AFG2	1.7
AFM1	0.71
OTA	0.4
OTα	10
GT	20
^13^C_17_-AFB1	0.6
^13^C_20_-OTA	0.6

## 5 Specimen collection, sample preparation, and solid-phase extraction

### 5.1 Specimen collection

Urine samples are collected in sealable polypropylene containers, aliquoted, and stored at −20 °C until sample preparation.

### 5.2 Sample preparation

Prior to sample preparation, the urine samples are brought to room temperature and homogenised. 4 ml of the urine sample are placed in a centrifuge tube (13 ml, round-bottom), mixed with 4 ml of ultra-pure water and 20 μl of the ISTD spiking solution and thoroughly mixed. The urine samples are subsequently centrifuged at 2045 × *g* and 10 °C for 15 min. The supernatant is then decanted into another centrifuge tube (13 ml, round-bottom).

### 5.3 Solid-phase extraction

The analytes are concentrated using Oasis HLB-cartridges. The cartridges are conditioned, first with 5 ml of methanol and then with 5 ml of diluted hydrochloric acid (55 mM, pH = 1.3). The samples are loaded portion by portion without applying a vacuum (flow rate approx. 1 ml/min) and the 13-ml centrifuge tubes are rinsed with 2 ml of ultra-pure water, which is also applied to the cartridges. After application of the sample, the cartridges are washed with 2 ml of 2% methanol in ultra-pure water. The cartridges are dried by briefly applying a vacuum (approx. 5 min). The cartridges must not run dry during the described conditioning of the cartridges and during sample application.

Using 2 × 2.5 ml of methanol, the analytes are eluted in centrifuge tubes (15 ml, conical base) into which 200 μl of ultra-pure water have been placed. The cartridges are briefly suctioned dry under a weak vacuum. Then, the eluates are blown down to about 200 μl at 40 °C under a stream of nitrogen. The concentrated eluates are then mixed with 300 μl of gradient solution, homogenised on a vortex shaker, and then filtered into polypropylene vials (1.5 ml, threaded) via syringe filters.

## 6 Operational parameters

Analytical determination was carried out using a device combination comprised of a liquid chromatograph (HPLC ­system: Nexera XR, Shimadzu Deutschland GmbH, Duisburg, Germany) and a tandem mass spectrometer (AB SCIEX QTRAP 5500, AB SCIEX Germany GmbH, Darmstadt, Germany).

### 6.1 High-performance liquid chromatography

**Table:** 

HPLC column:	Kinetex^®^ biphenyl; 2.6 μm; 100 × 2.1 mm
Precolumn:	UHPLC precolumn biphenyl 2.1 mm ID
Column-oven temperature:	40 °C
Autosampler temperature:	15 °C
Injection volume:	20 μl
Mobile phase A:	0.1% acetic acid and 1 mM ammonium acetate in ultra-pure water
Mobile phase B:	0.1% acetic acid and 1 mM ammonium acetate in methanol
Gradient programme:	see Table 5

**Tab.5:** Gradient programme for the determination of aflatoxins, ochratoxin A, free ochratoxin α, and gliotoxin in urine

Time [min]	Flow rate [ml/min]	Eluent A [%]	Eluent B [%]
0.01	0.45	98	2
2	0.45	98	2
5	0.45	20	80
5.2	0.45	2	98
8	0.45	2	98
8.01	0.45	98	2
11	0.45	98	2

### 6.2 Tandem mass spectrometry

**Table:** 

Source:	TurboSpray
Ionisation mode:	ESI, positive or negative
Ion-spray voltage:	5500 V or −4500 V
Source temperature:	500 °C
Nebulising gas:	Nitrogen, 80 psi
Turbo-heater gas:	Nitrogen, 80 psi
Curtain gas:	Nitrogen, 35 psi
Collision gas:	Nitrogen
Scan mode:	Multiple Reaction Monitoring (MRM)
Dwell time:	20 ms or 60 ms
Parameter-specific settings:	see Table 6

The instrument-specific parameters must be ascertained and adjusted by the user for the MS/MS system used. The device-specific parameters given in this section have been determined and optimised for the system used during method development.

Two mass transitions were selected for each analyte. One transition serves the purpose of quantification (quantifier) and the other of confirmation (qualifier). For the ISTDs two or three mass transitions were used. The selected transitions are summarised in Table 6 alongside the retention times and further MRM parameters. The retention times given below are only intended as a point of reference, the user must ensure the separation performance of the LC column used as well as the resulting retention behaviour of the substances.

**Tab.6:** Retention times and MRM parameters for the determination of aflatoxins, ochratoxin A, free ochratoxin α and gliotoxin in urine

Analyte/ISTD	Retention time [min]	Q1 (***m/z***)	Q3 (***m/z***)	DP [V]	EP [V]	CE [V]	CXP [V]
AFB1	7.2	313	285^a)^	100	10	31	18
			241	100	10	49	18
AFB2	7.06	315	287.2^a)^	96	10	37	18
			259.2	96	10	43	18
AFG1	6.95	329	243^a)^	80	10	37	12
			200	80	10	53	12
AFG2	6.82	331	313.2	111	10	35	18
			245.2^a)^	111	10	43	14
AFM1	6.66	329.07	272.9^a)^	91	10	33	16
			228.9	91	10	55	14
OTA	6.72	404	239^a)^	91	10	32	12
			102	91	10	105	12
^13^C_20_-OTA	6.72	424.1	377.2^a)^	44	10	19	34
			203.1	60	10	59	12
			250.1	44	10	31	33
^13^C_17_-AFB1	7.2	330.3	301.2^a)^	80	10	35	16
			284.3	80	10	47	16
OTα	5.78	255	167^a)^	−55	−10	−34	−11
			211.1	−55	−10	−22	−15
GT	6.32	325.1	261^a)^	−35	−10	−14	−14
			243.2	−35	−10	−22	−17

a) quantifier

CE: collision energy; CXP: cell-exit potential; DP: declustering potential; EP: entrance potential

## 7 Analytical determination

**Fig.2:**
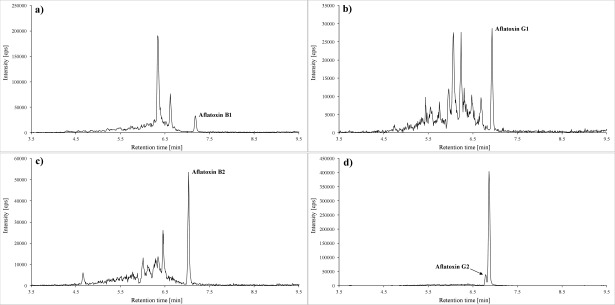
Quantifier transitions for a) aflatoxin B1 (0.013 μg/l; *m/z* 313 → 285), b) aflatoxin G1 (0.013 μg/l; *m/z* 329 → 243), c) afla­toxin B2 (0.022 μg/l; *m/z* 315 → 287.2) as well as for d) aflatoxin G2 (0.054 μg/l; *m/z* 331 → 245.2)

**Fig.3:**
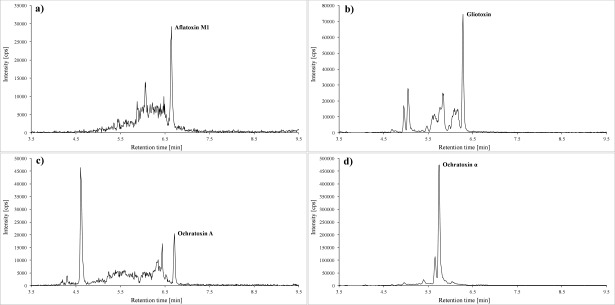
Quantifier transitions for a) aflatoxin M1 (0,022 μg/l; *m/z* 329 → 272,9), b) gliotoxin (1,5 μg/l; *m/z* 325,1 → 261), c) ochra­toxin A (0,013 μg/l; *m/z* 404 → 239) as well as d) ochratoxin α (1 μg/l; *m/z* 255 → 167)

Of each of the samples prepared according to Section 5, 20 μl are injected into the LC‑MS/MS system. The analytes are identified by their specific ion transitions and retention times. Figures 2 and 3 depict representative chromatograms of the calibration standard 1.

## 8 Calibration

The calibration solutions prepared according to Section 4.6 are processed in the same way as the samples (cf. Section 5, without addition of ISTD) and analysed by LC‑MS/MS (cf. Section 6).

**Fig.4:**
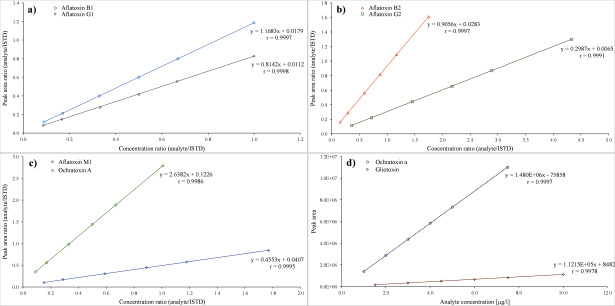
Calibration curves for the determination of a) aflatoxin B1 and aflatoxin G1, b) aflatoxin B2 and aflatoxin G2, c) aflatoxin M1 and ochratoxin A as well as d) free ochratoxin α and gliotoxin in urine (linear regression using 1/x-weighting)

The calibration curves for the aflatoxins and OTA are generated by plotting the quotients of the peak area of the analyte and the peak area of the isotope-labelled ISTD against the quotient of the spiked concentration of the analyte and the spiked concentration of the isotope-labelled ISTD. For the quantitation of aflatoxins and OTA, the peak areas are related to ^13^C_17_‑AFB1 and ^13^C_20_‑OTA, respectively. The calibration curves for OTα and GT are generated by plotting the peak areas against the respective spiked concentrations. A linear correlation with correlation coefficients of r ≥ 0.995 (1/x-weighting) was found for all analytes in the analysed concentration ranges. Figure 4 shows examples of calibration curves for the determination of aflatoxins as well as OTA, OTα and GT in urine.

## 9 Calculation of analytical results

To calculate the contents of the aflatoxins and OTA in a urine sample, the peak area of the individual analyte is divided by the peak area of the ISTD. With the calibration function corresponding to the analytical run in question, the quotient thus obtained can be used to calculate the analyte concentration in μg/l urine. For OTα and GT, the analyte concentration in μg/l urine is determined from the peak area using the calibration function corresponding to the analytical run in question.

## 10 Standardisation of measurement results and quality control

Quality assurance of the analytical results is carried out as stipulated in the guidelines of the *Bundesärzte­kammer* (German Medical Association) and in a general chapter published by the Commission (Bader et al. 2010; Bundesärztekammer 2014).

For quality control, blank and double-blank samples prepared in urine are measured as part of every calibration. Double-blank samples contain neither analyte nor ISTD. In contrast, blank samples are spiked with the ISTDs but are not spiked with the analyte. Additionally, a reagent blank (ultra-pure water instead of a urine sample) is processed and analysed as part of each analytical run.

To test for precision, each analytical run includes at least two quality-control samples with a known concentration of the analytes. Since commercial material is not available, the control material must be prepared in the in-house laboratory by spiking urine with standard solutions of the analytes in the relevant concentration range (see Tables 7 and 8). In addition, the control-standard solution (see Section 4.7) and unspiked gradient solution are analysed in each run and washing steps with methanol are performed.

**Tab.7:** Pipetting scheme for the preparation of quality-control samples

Solution	S 1 [μl]	S 2 [μl]	S 3 [μl]	ISTD-spiking solution [μl]	Urine [μl]
Q_low_	7.5	20	15	20	4000
Q_high_	25	50	35	20	4000

**Tab.8:** Concentration of the analytes and ISTDs in the quality-control samples

Solution	Concentration [μg/l]									
	AFB1	AFB2	AFG1	AFG2	AFM1	OTA	OTα	GT	^13^C_17_-AFB1	^13^C_20_-OTA
Q_low_	0.0375	0.0654	0.0375	0.163	0.066	0.0377	2.5	3.75	0.15	0.15
Q_high_	0.13	0.22	0.13	0.54	0.22	0.13	6.25	8.75	0.15	0.15

## 11 Evaluation of the method

The reliability of this method was confirmed by comprehensive validation as well as by replication and verification in a second, independent laboratory.

The verifiers of the method additionally included the mycotoxin citrinin and its metabolite DH-CIT into the method. The validation data for these two parameters are given in the Appendix, but they were not independently verified.

### 11.1 Precision

To determine within-day precision, the prepared quality-control samples Q_low_ and Q_high_ were processed and analysed six times in parallel. The precision data thus obtained are presented in Table 9.

**Tab.9:** Within-day precision for the determination of the aflatoxins, ochratoxin A, free ochratoxin α, and gliotoxin in urine

Analyte	Spiked concentration [μg/l]	Number ***n***	Measured concentration [μg/l]	Standard deviation (rel.) ***s_w_*** [%]	Prognostic range ***u*** [%]
AFB1	0.0375	6	0.0370	2.9	7.4
	0.13	6	0.1302	1.8	4.6
AFB2	0.0654	6	0.0683	2.4	6.2
	0.22	6	0.2239	2.2	5.6
AFG1	0.0375	6	0.0373	3.5	9.1
	0.13	6	0.1303	2.6	6.8
AFG2	0.163	6	0.1636	2.0	5.0
	0.54	6	0.5549	3.2	8.3
AFM1	0.066	6	0.0714	3.1	7.9
	0.22	6	0.2218	3.6	9.4
OTA	0.0377	6	0.0390	1.5	3.9
	0.13	6	0.1216	3.6	9.4
OTα	2.5	6	2.38	1.3	3.3
	6.25	6	5.84	4.0	10.4
GT	3.75	6	3.58	1.7	4.4
	8.75	6	8.13	4.9	12.7

To determine day-to-day precision, control materials Q_low_ and Q_high_ were processed and analysed in duplicate on six to eight days, depending on the analyte. The precision data thus obtained are presented in Table 10.

**Tab.10:** Day-to-day precision for the determination of the aflatoxins, ochratoxin A, free ochratoxin α, and gliotoxin in urine

Analyte	Spiked concentration [μg/l]	Number ***n***	Measured concentration [μg/l]	Standard deviation (rel.) ***s_w_*** [%]	Prognostic range ***u*** [%]
AFB1	0.0375	6	0.0396	5.2	13.4
	0.13	8	0.1215	3.6	8.6
AFB2	0.0654	6	0.0673	2.2	5.6
	0.22	8	0.2106	3.6	8.5
AFG1	0.0375	6	0.0399	4.9	12.5
	0.13	8	0.1231	3.5	8.3
AFG2	0.163	6	0.1562	2.7	7.0
	0.54	8	0.5200	3.3	7.8
AFM1	0.066	6	0.0681	3.6	9.3
	0.22	8	0.2121	5.9	13.9
OTA	0.0377	6	0.0349	8.8	22.7
	0.13	7	0.1077	12.5	30.6
OTα	2.5	6	2.50	5.2	13.2
	6.25	8	6.22	6.5	15.3
GT	3.75	6	3.64	6.6	16.9
	8.75	8	9.08	8.6	20.4

### 11.2 Accuracy

The accuracy of the method was determined from the within-day and day-to-day precision. The calculated mean relative recoveries for the individual analytes are given in Table 11.

**Tab.11:** Mean relative recoveries for the determination of the aflatoxins, ochratoxin A, free ochratoxin α, and gliotoxin in urine

Analyte	Spiked concentration [μg/l]	Within-day precision			Day-to-day precision		
		Number ***n***	Recovery (rel.) ***r*** [%]	Range [%]	Number ***n***	Recovery (rel.) ***r*** [%]	Range [%]
AFB1	0.0375	6	98.8	95.4–103	6	106	98.8–113
	0.13	6	100	98.0–103	8	93.5	88.2–100
AFB2	0.0654	6	104	99.8–107	6	103	99.7–105
	0.22	6	102	98.3–104	8	95.7	91.8–102
AFG1	0.0375	6	99.4	94.8–104	6	107	99.4–112
	0.13	6	100	97.7–104	8	94.6	89.9–100
AFG2	0.163	6	100	97.4–103	6	95.8	92.6–100
	0.54	6	103	97.5–107	8	96.3	93.1–103
AFM1	0.066	6	108	105–114	6	103	98.1–108
	0.22	6	101	95.5–106	8	96.4	86.9–105
OTA	0.0377	6	103	102–105	6	92.5	84.0–103
	0.13	6	93.5	87.8–96.3	7	82.9	70.4–96.0
OTα	2.5	6	95.4	94.3–97.5	6	99.9	91.6–104
	6.25	6	93.4	90.3–100	8	99.5	93.4–112
GT	3.75	6	95.4	93.2–97.2	6	97.1	90.8–108
	8.75	6	92.9	86.8–97.1	8	104	92.9–117

### 11.3 Absolute recovery

The verifiers of the method determined the losses due to processing. For this purpose, urine samples were spiked with the analytes and the ISTDs before processing (series A) or spiked with the maximum expected amounts of the analytes after sample preparation (series B). The quotients of the peak areas of sample series A and sample series B represent the analyte losses due to sample preparation and are summarised for the concentrations investigated in Table 12.

**Tab.12:** Absolute recoveries for the determination of the aflatoxins, ochratoxin A, free ochratoxin α, and gliotoxin in urine (n = 6)

Analyte	Spiked concentration [µg/l]	Peak area ratio series A/series B
AFB1	0.01	0.91
	0.0503	0.94
	0.1005	0.91
AFB2	0.0176	0.96
	0.0878	0.96
	0.1757	0.93
AFG1	0.01	0.87
	0.0508	0.92
	0.1015	0.86
AFG2	0.0426	0.97
	0.2129	0.94
	0.4259	0.90
AFM1	0.0175	0.91
	0.0877	0.95
	0.1754	0.91
OTA	0.01	0.90
	0.0502	0.95
	0.1003	0.90
OTα	0.77	0.85
	3.06	1.05
	5.1	1.02
GT	1.0	0.73
	4.5	0.83
	7.5	0.90

### 11.4 Limits of detection and quantitation

The detection limits given in Table 13 were estimated from the threefold signal-to-noise ratio and the quantitation limits from the tenfold signal-to-noise ratio. 

**Tab.13:** Limits of detection and quantitation for the determination of the aflatoxins, ochratoxin A, free ochratoxin α, and gliotoxin in urine

Analyte	Detection limit [μg/l]	Quantitation limit [μg/l]
AFB1	0.004	0.013
AFB2	0.007	0.022
AFG1	0.004	0.013
AFG2	0.02	0.054
AFM1	0.007	0.022
OTA	0.004	0.013
OTα	0.4	1.0
GT	0.5	1.5

### 11.5 Analyte stability in the urine matrix

Analyte stability in the urine matrix was investigated at room temperature, at 4 °C, and at −20 °C. Stability at room temperature, which is relevant for sample preparation, was examined over a period of 24 hours. Stability during refrigerated storage at 4 °C, which is relevant for short-term storage of urine samples, was examined over a period of 48 hours. Stability at −20 °C is relevant for longer sample storage and was determined after one week, two weeks, and four and a half weeks.

To determine analyte stability, Q_low_ and Q_high_ samples were processed and analysed in duplicate. Acceptance criteria were based on Commission Decision 2002/657/EC of the European Union, which allows for a deviation from the nominal value of −50 to +20% (European Commission 2002).

For storage at room temperature as well as at 4 °C and at −20 °C, the recoveries of the analytes in the urine matrix were found to be within the accepted range. The data are given in Table 14.

**Tab.14:** Recoveries of the analytes after storage at room temperature, 4 °C, and −20 °C

Analyte	Spiked concentration [μg/l]	Room temperature	4 °C	−20 °C	−20 °C	−20 °C
		Recovery after 24 h [%]	Recovery after 48 h [%]	Recovery after 1 week [%]	Recovery after 2 weeks [%]	Recovery after 4.5 weeks [%]
AFB1	0.0375	107	110	101	90.6	91.8
	0.13	104	108	99.2	91.6	102
AFB2	0.0654	102	104	103	90.4	99.0
	0.22	99.6	102	97.8	91.3	110
AFG1	0.0375	103	112	83.6	72.6	110
	0.13	115	113	82.7	78.6	118
AFG2	0.163	95.8	98.0	92.6	88.2	98.2
	0.54	99.1	104	92.0	89.9	108
AFM1	0.066	96.8	103	86.9	84.1	118
	0.22	100	100	80.6	80.1	107
OTA	0.0377	82.7	88.8	106	102	91.4^a)^
	0.13	82.2	79.1	98.8	88.5	94.7^a)^
OTα	2.5	95.1	99.6	90.8	80.0	90.2
	6.25	88.9	81.2	87.4	92.8	110
GT	3.75	84.9	85.3	119	99.0	80.5^a)^
	8.75	92.8	91.8	113	112	83.2^a)^

a) recovery after 5 weeks

### 11.6 Analyte stability in the ready-to-measure samples

Analyte stability in the extract of the processed quality-control samples was determined after storage at −20 °C for one and four weeks. Again acceptance criteria were based on Commission Decision 2002/657/EC of the European Union (European Commission 2002). Table 15 presents the results of analyte stability in the ready‑to‑measure samples. After storage at −20 °C, the recoveries of the analytes in the extracts were between 80.8% and 105%.

**Tab.15:** Analyte stability in the ready-to-measure samples after storage at −20 °C

Analyte	Spiked concentration [μg/l]	Recovery after 1 week [%]	Recovery after 4 weeks [%]
AFB1	0.0375	105	97.4
	0.13	104	100
AFB2	0.0654	102	101
	0.22	104	104
AFG1	0.0375	82.3	108
	0.13	80.8	105
AFG2	0.163	96.6	108
	0.54	98.8	104
AFM1	0.066	94.5	114
	0.22	88.2	111
OTA	0.0377	105	101^a)^
	0.13	98.4	106^a)^
OTα	2.5	95.0	96.0
	6.25	90.1	110
GT	3.75	102	105^a)^
	8.75	109	105^a)^

a) recovery after 2 weeks

### 11.7 Sources of error

To prepare the calibration standards, urines from various individuals were tested for the concentrations of the analytes to be measured. As non-negligible concentrations of OTA were found in almost all of the tested urine samples, pool urine could not be used. The calibration standards were finally prepared in an almost uncontaminated native urine, which was collected in larger amounts and stored at −20 °C.

## 12 Discussion of the method

This method enables the reliable determination of aflatoxins, OTA, free OTα, and GT in urine. The validation data demonstrate the good reproducibility, accuracy, and sensitivity of the method. The quantitation limit for OTA determined by the developers of the method is similar to the limits of quantitation of other methods (Föllmann et al. 2016). At 1 μg/l, the quantitation limit for OTα exceeded values indicated in other studies (Njumbe Ediage et al. 2012). No data from other studies were available for GT. For the aflatoxins, the limits of quantification determined during method development were in part similar to those of other methods (Gerding et al. 2015; Schmidt et al. 2021), but were in part also below the values specified in other studies (Penczynski et al. 2022; Schmidt et al. 2021). The limits of quantitation obtained during external verification of the method were even lower, which is probably due to the use of a more sensitive tandem mass spectrometer.

As part of method development, various SPE materials for purification and enrichment were tested as well as various HPLC separation columns and eluent mixtures for HPLC. As far as the analytical column is concerned, the Kinetex biphenyl column (Phenomenex) was the only one that led to a satisfactory separation of the mycotoxins. Moreover, with other RP columns it was not possible to separate the analytes from the urine matrix.

The aflatoxins were evaluated using the ISTD ^13^C_17_‑AFB1. At the beginning of method development, further ISTDs (^13^C_17_‑AFB2 and ^13^C_17_‑AFM1) were used. However, comprehensive tests showed that the ISTD ^13^C_17_‑AFB1 could be applied for the quantitation of all investigated aflatoxins. OTA was analysed using ^13^C_20_‑OTA. OTα and GT were determined without the use of an ISTD.

During the method development, the concentrations of the calibration standards were adjusted to real samples from an ongoing study; as a result, the calibration ranges of the individual analytes were decreased. The concentrations of the ISTDs were not changed so that users of the method can apply them in lower concentrations if necessary.

The investigation of analyte stability in the urine matrix and in the ready-to-measure samples confirmed the instability of GT in urine already described in the literature (Cerqueira et al. 2014), so that the solutions for GT should be freshly prepared every week.

The verifiers of the method applied it to analyse 32 urine samples (24-h urines) taken from 16 raw foodists and 16 control persons (vegans and omnivores). Quantifiable amounts of OTA (94%) and CIT (94%) were detected in most of the samples. Compared to the control group, lower amounts of OTA and CIT were found in the urine of the raw foodists. The aflatoxins, OTα and free GT were not detected in any of the samples.

**Instruments used** HPLC system with a binary pump, autosampler, column oven, and degasser (Nexera XR, Shimadzu Deutschland GmbH, Duisburg, Germany); triple-quadrupole mass spectrometer (Model AB SCIEX QTRAP 5500 with electrospray ionisation, AB SCIEX Germany GmbH, Darmstadt, Germany)

## Figures and Tables

**Tab.16:** Pipetting scheme for the preparation of the calibration solutions for the determination of citrinin and dihydrocitrinone in urine

Solution	CIT and DH-CIT spiking solution [μl]	CIT concentration [µg/l]	DH-CIT concentration [µg/l]	^13^C_13_-CIT spiking solution [μl]	^13^C_13_-CIT concentration [μg/l]
DB^a)^	0	0	0	0	0
B^b)^	0	0	0	20	1.017
1	4^c)^	0.001	0.001	20	1.017
2	10^c)^	0.0025	0.0025	20	1.017
3	20^c)^	0.005	0.005	20	1.017
4	30c)	0.0075	0.0075	20	1.017
5	40^c)^	0.01	0.01	20	1.017
6	100^c)^	0.025	0.025	20	1.017
7	4	0.05	0.05	20	1.017
8	8	0.1	0.1	20	1.017
9	40	0.5	0.5	20	1.017
10	80	1.001	1	20	1.017
11	160	2.002	2	20	1.017

a) double-blank

b) blank

c) previous dilution step: 20 μl of the spiking solution with 980 μl of acetonitrile

**Tab.17:** Concentrations of analytes in the control-standard solution

Analyte/ISTD	Concentration [μg/l]
CIT	1.001
DH-CIT	1.0
^13^C_13_-CIT	2.034

**Table:** 

HPLC column:	Kinetex^®^ biphenyl; 2.6 μm; 100 × 2.1 mm
Precolumn:	UHPLC precolumn biphenyl; 2.1 mm ID
Column-oven temperature:	40 °C
Autosampler temperature:	10 °C
Injection volume:	10 μl
Mobile phase A:	0.1% acetic acid and 1 mM ammonium acetate in ultra-pure water
Mobile phase B:	0.1% acetic acid and 1 mM ammonium acetate in methanol
Gradient programme:	see Table 18

**Tab.18:** Gradient programme for the determination of citrinin and dihydrocitrinone in urine

Time [min]	Flow rate [ml/min]	Eluent A [%]	Eluent B [%]
0	0.45	95	5
1	0.45	95	5
8	0.45	2	98
9	0.45	2	98
9.01	0.45	95	5
11	0.45	95	5

**Table:** 

Source:	TurboSpray
Ionisation mode:	ESI negative
Ion-spray voltage:	−4500 V
Source temperature:	450 °C
Nebulising gas:	Nitrogen, 60 psi
Turbo-heater gas:	Nitrogen, 50 psi
Curtain gas:	Nitrogen, 40 psi
Collision gas:	Nitrogen
Scan mode:	Multiple Reaction Monitoring (MRM)
Parameter-specific settings:	see Table 19

**Tab.19:** Retention times and MRM parameters for the determination of citrinin and dihydrocitrinone in urine

Analyte/ISTD	Retention time [min]	Q1 (***m/z***)	Q3 (***m/z***)	Dwell time [msec]	DP [V]	CE [V]	CXP [V]
CIT	5.23	280.9 [M-H+CH_3_OH]^–^	248.9^a)^	40	−60	−22	−15
			204.9	40	−60	−32	−11
		248.9 [M-H]^–^	176.9	40	−150	−30	−11
DH-CIT	5.13	264.9 [M-H]^–^	220.9^a)^	40	−50	−30	−13
			246.9	40	−50	−30	−15
			176.9	40	−50	−34	−17
^13^C_13_-CIT	5.23	293.99 [M-H+CH_3_OH]^–^	261.96^a)^	40	−40	−24	−7
			217.0	40	−40	−32	−21

a) quantifier

CE: collision energy; CXP: cell-exit potential; DP: declustering potential

**Fig.5:**
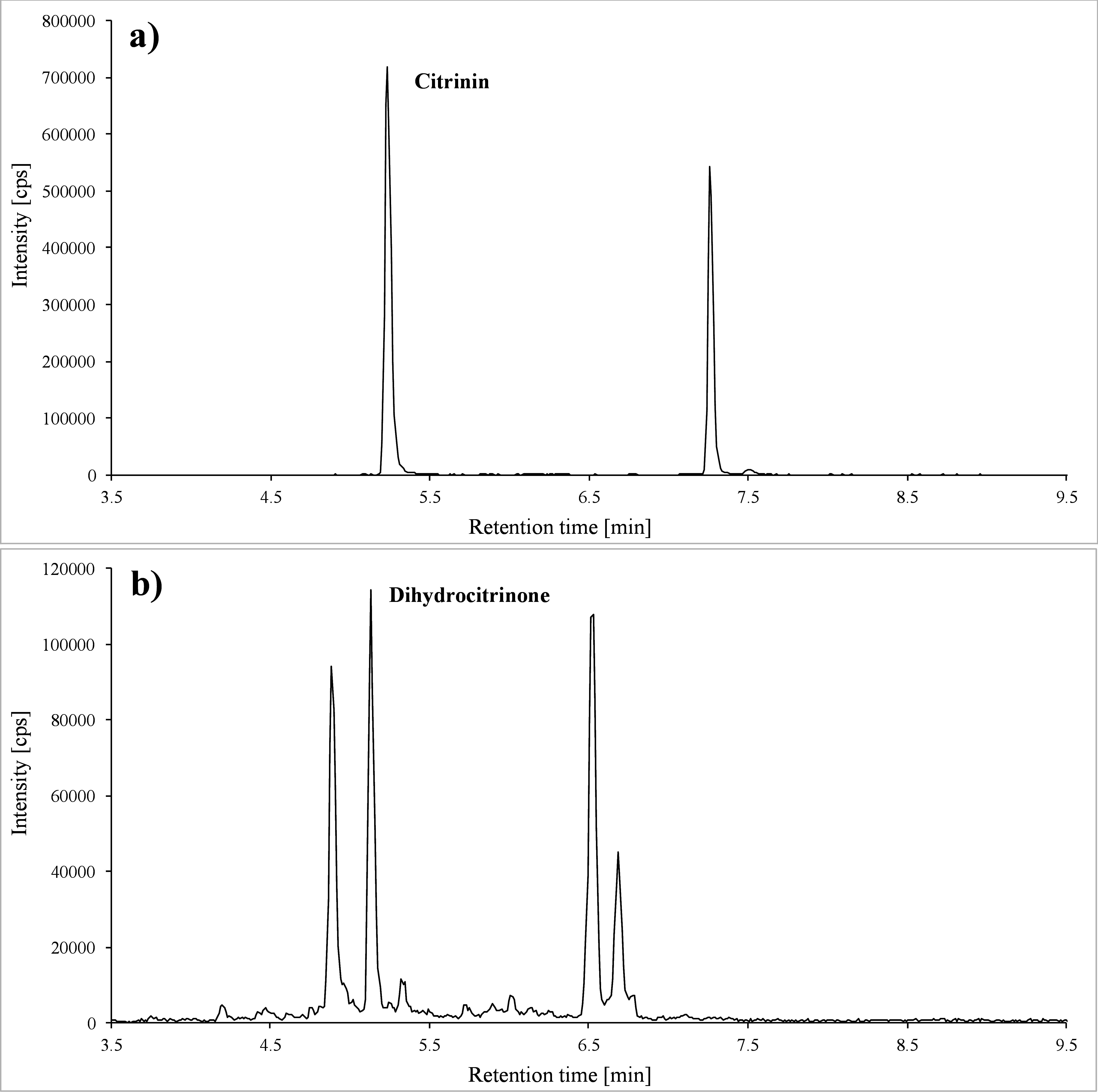
Chromatograms of a blank urine spiked with a) 0.5 μg citrinin/l (quantifier *m/z* 281 → 249) and with b) 0.5 μg dihydrocitrinone/l (quantifier *m/z* 265 → 221)

**Fig.6:**
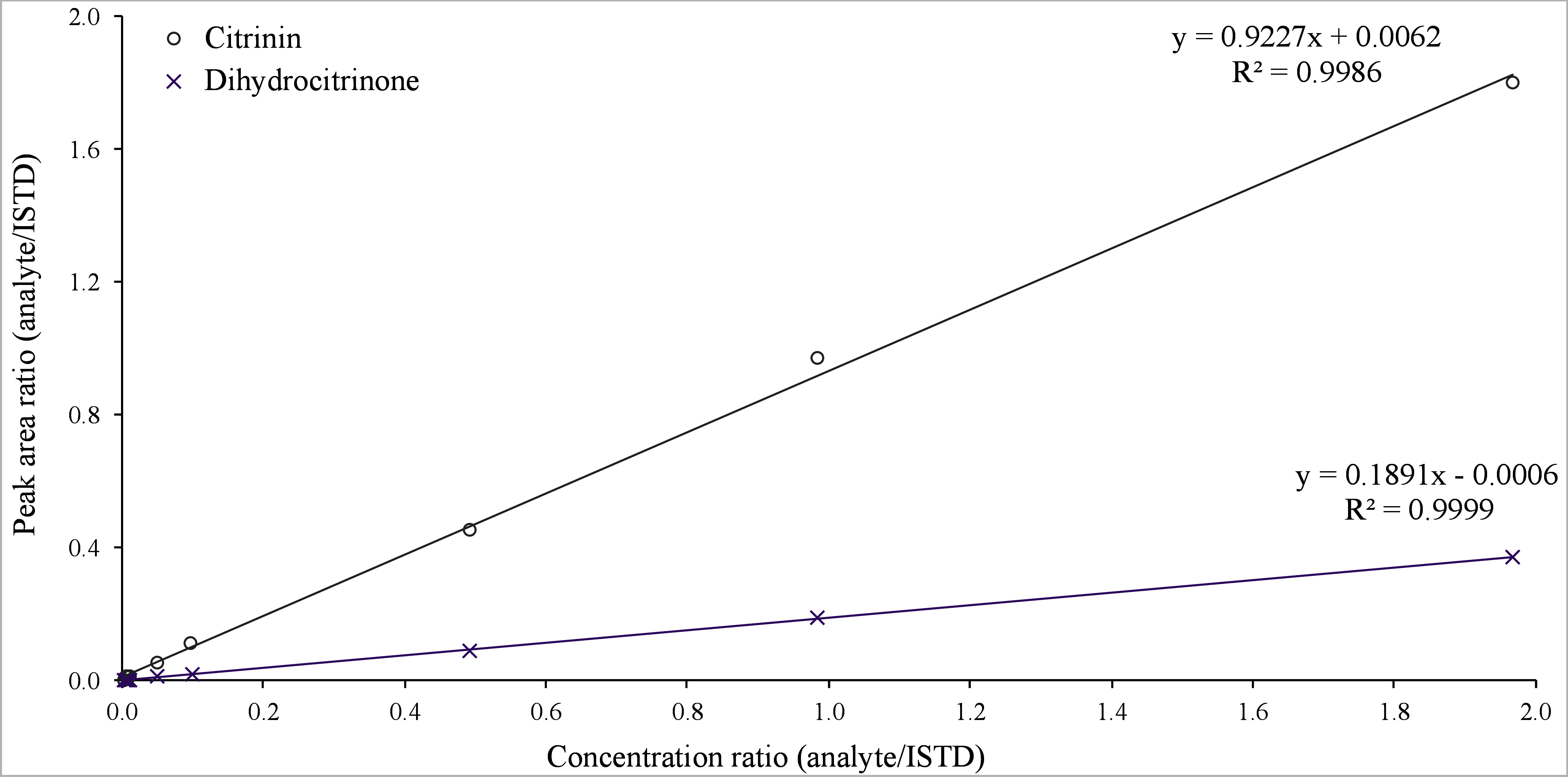
Calibration curve for the determination of citrinin and dihydrocitrinone (linear regression without weighting)

**Tab.20:** Within-day precision for the determination citrinin and dihydrocitrinone in urine

Analyte	Spiked concentration [μg/l]	Number ***n***	Measured concentration [μg/l]	Standard deviation (rel.) ***s_w_*** [%]	Prognostic range ***u*** [%]
CIT	0.01	6	0.01	3.1	9.4
	0.1	6	0.09	6.0	16.5
	1.0	6	0.93	6.5	17.2
DH‑CIT	0.01	6	0.0108	14.0	37.5
	0.1	6	0.08	3.3	10.1
	1.0	6	0.89	8.9	23.9

**Tab.21:** Day-to-day precision for the determination of citrinin and dihydrocitrinone in urine

Analyte	Spiked concentration [μg/l]	Number ***n***	Measured concentration [μg/l]	Standard deviation (rel.) ***s_w_*** [%]	Prognostic range ***u*** [%]
CIT	0.01	6	0.01	6.4	17.5
	0.1	6	0.10	5.9	15.2
	1.0	6	0.98	2.1	6.2
DH‑CIT	0.01	6	0.01	19.3	54.7
	0.1	6	0.09	5.4	12.1
	1.0	6	0.96	3.4	9.9

**Tab.22:** Mean relative recoveries for the determination of citrinin and dihydrocitrinone in urine

Analyte	Spiked concentration [μg/l]	Within-day precision			Day-to-day precision		
		Number ***n***	Recovery (rel.) ***r*** [%]	Range [%]	Number ***n***	Recovery (rel.) ***r*** [%]	Range [%]
CIT	0.01	6	94.1	89.3–98.2	6	99.8	90.4–108
	0.1	6	88.6	79.7–94.3	6	99.0	91.2–106
	1.0	6	93.3	89.1–105	6	98.5	89.1–105
DH‑CIT	0.01	6	108	93.0–134	6	108	75.9–135
	0.1	6	80.9	77.0–85.2	6	88.4	82.3–93.1
	1.0	6	88.5	81.2–102	6	96.4	90.7–100

**Tab.23:** Absolute recoveries for the determination of citrinin and dihydrocitrinone in urine (n = 6)

Analyte	Spiked concentration [µg/l]	Peak area ratio series A/series B
CIT	0.01	1.03
	0.1	0.93
	1.0	0.99
DH-CIT	0.01	1.04
	0.1	0.95
	1.0	1.01

**Tab.24:** Limits of detection and quantitation for the determination of citrinin and dihydrocitrinone in urine

Analyte	Blank urine		Pooled urine	
	Detection limit [μg/l]	Quantitation limit [μg/l]	Detection limit [μg/l]	Quantitation limit [μg/l]
CIT	0.0003^a)^	0.001^a)^	0.01^b)^	0.025^b)^
DH-CIT	0.0075	0.01	0.5^b)^	0.5^b)^

a) The blank urine contained low background levels of citrinin. The S/N values of all samples (abscissa) were plotted against the nominal concentrations (ordinate) to estimate the limits of detection and quantification. After subtracting the mean S/N value from unspiked blank urine samples, the limits of detection and quantitation were estimated from the resulting regression functions and confirmed visually.

b) The pooled urine contained low background levels of citrinin and dihydrocitrinone. The S/N values of all samples (abscissa) were plotted against the nominal concentrations (ordinate) to estimate the limits of detection and quantification. After subtracting the mean S/N value from unspiked pooled urine samples, the limits of detection and quantitation were estimated from the resulting regression functions and confirmed visually.

## Appendix

During external verification CIT and its metabolite DH-CIT – in addition to the aflatoxins, OTA, free OTα, and GT – were included in the method. The general procedure corresponded to the described method of the developers, so that only the information required for the quantification of CIT and DH-CIT is presented here. The validation data collected by the verifiers of the method are listed below, although these were not independently verified.

### Equipment, chemicals, and solutions

#### Equipment

- UPLC system with a binary pump and column oven (e.g. Acquity UPLC I-Class with SM-FTN Sample Manager, Waters GmbH, Eschborn, Germany)
- Tandem mass spectrometer (e.g. AB SCIEX QTRAP 6500, AB SCIEX Germany GmbH, Darmstadt, Germany)

#### Reference standards and ISTD

- Citrinin, 100.1 µg/ml in acetonitrile (e.g. Romer Labs Division Holding GmbH, Getzersdorf, Austria)
- Dihydrocitrinone (e.g. AnalytiCon Discovery GmbH, Potsdam, Germany)
- ^13^C_13_‑Citrinin, 10.17 µg/ml in acetonitrile (e.g. Romer Labs Division Holding GmbH, Getzersdorf, Austria)

#### Solutions – reference standards and ISTD

- ^13^C_13_-CIT spiking solution (203.4 μg ^13^C_13_-CIT/l)
   20 μl of the ^13^C_13_-CIT stock solution are mixed with 980 μl of acetonitrile in a 1.5‑ml polypropylene vial.

The ^13^C_13_-CIT spiking solution was stored at –80 °C. At this storage temperature, the analyte was stable over a period of twelve weeks.

- CIT working solution (1.001 mg/l)
   100 μl of the CIT stock solution are pipetted into a 10‑ml volumetric flask, which is then made up to the mark with acetonitrile.

The CIT working solution is stored at –80 °C and is stable for three months.

- DH-CIT working solution I (500 mg/l)
   5 mg of DH-CIT are weighed into a 10‑ml volumetric flask and dissolved in a little acetonitrile. Subsequently, the volumetric flask is made up to the mark with acetonitrile.

The DH-CIT working solution I is stored at –80 °C and is stable for three months.

- DH-CIT working solution II (5 mg/l)
   100 μl of the DH-CIT working solution I are pipetted into a 10‑ml volumetric flask. Subsequently, the volumetric flask is made up to the mark with acetonitrile.

The DH-CIT working solution II is freshly prepared for each calibration as well as for the preparation of the quality-control samples.

- CIT and DH-CIT spiking solution (50.05 μg CIT/l and 50.0 μg DH-CIT/l)
   50 μl of the CIT working solution and 10 μl of the DH-CIT working solution II are mixed with 940 μl of acetonitrile in a 1.5‑ml polypropylene vial.

The CIT and DH-CIT spiking solution is freshly prepared for each calibration as well as for the preparation of the quali­ty-control samples.

#### Calibration standards

During method verification, the calibration solutions were prepared in almost uncontaminated native urine. As urine samples normally contain measurable levels of various mycotoxins, urine was collected from a person who had abstained from certain foods (cereal products, rice, dairy products, spices, wine, coffee) for five days. The mycotoxin levels in this urine were generally below the detection limits.

To prepare the calibration standards, the verifiers of the method pipetted the spiking solutions into 4 ml of urine according to the pipetting scheme shown in Table 16. For the analyte concentrations in the range from 0.001 μg/l to 0.025 μg/l, the CIT and DH-CIT spiking solution was further diluted.

The calibration solutions are prepared in the same way as the samples as described in Section 5, but without the addition of ISTD.

The calibration standards are designed to determine the linear range of detection as well as the limits of detection and quantitation. All eleven solutions are used for calibration, whereby the concentrations below the limit of quantification of the respective analyte are not included in the calibration.

#### Control-standard solution

The control‑standard solution was prepared by diluting the CIT and DH-CIT spiking solution with gradient solution, aliquoted in 250 μl volumes and stored at –80 °C. Table 17 shows the CIT and DH-CIT concentrations in this solution.

### Analytical determination

Of each of the samples prepared 10 μl are injected into the LC‑MS/MS system. Figure 5 depicts representative chromato­grams for CIT and DH-CIT.

### Calibration

To generate the calibration curves for the determination of CIT and DH-CIT, the peak areas of the analytes are related to the ^13^C_13_‑CIT-peak area and plotted against the quotient of the spiked concentration of the analyte and the spiked concentration of the ISTD. For CIT and DH-CIT, a linear correlation with coefficients of determination of R^2^ ≥ 0.998 was obtained in the analysed concentration ranges. Figure 6 shows calibration curves for the determination of CIT and DH-CIT in urine as examples.

### Calculation of analytical results

To calculate the CIT or DH-CIT content of a sample, the peak area of the analyte is divided by the peak area of ^13^C_13_‑CIT. The analyte content in μg/l urine can be calculated from the quotient determined using the calibration function corresponding to the analytical run in question.

### Standardisation and quality control

In the three control materials used in the external verification, the concentrations for CIT and DH-CIT were 0.01 μg/l, 0.1 μg/l, and 1 μg/l, respectively.

### Evaluation of the method

The validation data for CIT and DH-CIT were not independently verified.

#### Precision

For CIT and DH-CIT, the within-day precision was determined by the verifiers of the method by preparing and analysing the three control materials six times in parallel.

The day-to-day precision was determined by processing and analysing the three control materials on six days.

#### Accuracy

The accuracy of the method was determined from the within-day and day-to-day precision data.

#### Absolute recovery

The losses due to processing were also determined for CIT and DH-CIT. For this purpose, urine samples were spiked with the analytes and the ISTDs before processing (series A) or spiked with the maximum expected amounts of the analytes after sample preparation (series B). The quotients of the peak areas of sample series A and sample series B represent the analyte loss due to sample preparation.

#### Limits of detection and quantitation

The detection and quantitation limits for the determination of CIT and DH-CIT were calculated from the threefold and tenfold signal-to-noise ratios, respectively.

The matrix-poor blank urine in combination with a more sensitive tandem mass spectrometer (AB SCIEX QTRAP 6500 instead of AB SCIEX QTRAP 5500) led to very low detection and quantitation limits. In a urine sample pooled from five individual urines, the limits of detection and quantitation were higher (Table 24).

### Sources of error and discussion

CIT and DH-CIT can be sensitively and reliably determined in human urine using the method described. The limits of quantitation of 0.025 µg CIT/l of urine and 0.5 µg DH-CIT/l of urine obtained using pooled urine allow for the quantitation of dietary background levels in the general population.

The validation data for CIT and DH-CIT show good reproducibility, accuracy and sensitivity of the method. The standard deviations for both the within-day precision and the precision from day to day are below 10% (for all analyte concentrations above the respective limit of quantification). Standard deviations of 14.0% and 19.3% were obtained for the DH-CIT standard spiked at the limit of quantitation (0.01 µg DH-CIT per litre).

The mean relative recoveries calculated from the precision data were between 80.9 and 108% and can therefore be described as very good. In addition, the absolute recovery shows that there are no losses of analyte due to sample preparation.

**Instruments used **UPLC system with a binary pump and column oven (e.g. Acquity UPLC I-Class with SM-FTN Sample Manager, Waters GmbH, Eschborn, Germany); tandem mass spectrometer (e.g. AB SCIEX QTRAP 6500, AB SCIEX Germany GmbH, Darmstadt, Germany)
